# Efficient one-step production of (*S*)-1-phenyl-1,2-ethanediol from (*R*)-enantiomer plus NAD^+^–NADPH *in*-*situ* regeneration using engineered *Escherichia coli*

**DOI:** 10.1186/1475-2859-11-167

**Published:** 2012-12-29

**Authors:** Rongzhen Zhang, Yan Xu, Rong Xiao, Botao Zhang, Lei Wang

**Affiliations:** 1 Key Laboratory of Industrial Biotechnology of Ministry of Education & School of Biotechnology, Jiangnan University, 1800 Lihu Avenue, Wuxi, 214122, P. R. China; 2 National Key Laboratory for Food Science, Jiangnan University, Wuxi, 214122, P. R. China; 3 Center for Advanced Biotechnology and Medicine, Rutgers University, Piscataway, NJ, 08854, USA

**Keywords:** Redox cofactor regeneration, Chiral alcohol, Alcohol dehydrogenases/reductases, Metabolic pathway, One-step stereoinversion

## Abstract

**Background:**

*Candida parapsilosis* CCTCC M203011 catalyzes the stereoinversion of (*R*)-1-phenyl-1,2-ethanediol (PED) through oxidation and reduction. Its NAD^+^-linked (*R*)-carbonyl reductase (RCR) catalyzes the oxidization of (*R*)-PED to 2-hydroxyacetophenone (HAP), and its NADPH-dependent (*S*)-carbonyl reductase (SCR) catalyzes the reduction of HAP to (*S*)-PED. The reactions require NAD^+^ and NADPH as cofactors. However, even if NAD^+^ and NADPH are added, the biotransformation of (*S*)-PED from the (*R*)-enantiomer by an *Escherichia coli* strain co-expressing RCR and SCR is slow and gives low yields, probably as a result of insufficient or imbalanced redox cofactors. To prepare (*S*)-PED from the (*R*)-enantiomer in one-step efficiently, plus redox cofactor regeneration, we introduced pyridine nucleotide transhydrogenases (PNTs) from *E*. *coli* to the metabolic pathway of (*S*)-PED.

**Results:**

The PNTs were successfully introduced into the *E*. *coli* strain RSAB. Most of the PNT activities occurred in the cell membrane of *E*. *coli*. The introduction of PNTs increased intracellular NAD^+^ and NADH concentrations and decreased the NADPH pool without affecting the total nucleotide concentration and cell growth properties. The presence of PNTs increased the NADH/NAD^+^ ratio slightly and reduced the NADPH/NADP^+^ ratio about two-fold; the ratio of NADPH/NADP^+^ to NADH/NAD^+^ was reduced from 36 to 17. So, the PNTs rebalanced the cofactor pathways: the rate of RCR was increased, while the rate of SCR was decreased. When the ratio of NAD^+^/NADPH was 3.0 or higher, the RSAB strain produced (*S*)-PED with the highest optical purity, 97.4%, and a yield of 95.2% at 6 h. The introduction of PNTs stimulated increases of 51.5% and 80.6%, respectively, in optical purity and yield, and simultaneously reduced the reaction time seven-fold.

**Conclusions:**

In this work, PNTs were introduced into *E*. *coli* to rebalance the cofactor pools within the engineered (*S*)-PED pathways. The efficient one-step production of (*S*)-PED plus NAD^+^–NADPH *in*-*situ* regeneration was realized. This work provided new insights into cofactor rebalancing pathways, using metabolic engineering methods, for efficient chiral alcohol production.

## Background

Alcohol dehydrogenases can catalyze a great variety of reduction–oxidation (redox) reactions during the production of chiral compounds [1-4]. However, their practical applications can be quite challenging since they require expensive cofactors, such as nicotinamide adenine dinucleotide [NAD^+^ and NADH] and nicotinamide adenine dinucleotide phosphate [NADP^+^ and NADPH] [5-7]. To address redox reaction limitations, enzyme-mediated cofactor recycling is preferred for industrial processes because of its high selectivity and efficiency [8,9]. For instance, Verho *et al*. improved pentose fermentation in *Saccharomyces cerevisiae* by engineering redox cofactor regeneration through the corresponding fungal pathways [10].

In general, either the cofactor couple NADH/NAD^+^ or NADPH/NADP^+^ is required in a specific biochemical reaction [9,10]. However, if both NAD(H) (NAD^+^ and NADH) and NADP(H) (NADP^+^ and NADPH) are required in a particular cofactor-dependent pathway, the flux is controlled not only by the availability of enzymes, but also by the cofactor amount and the ratio of the reduced forms to the oxidized forms of the cofactor [5,7,11]. The different cofactors have to be regenerated in separate processes and their intracellular redox forms need to be balanced to achieve high yields [5,12]. Several groups achieved the simultaneous regeneration of NAD(H) and NADP(H) in redox reaction systems by the introduction of pyridine nucleotide transhydrogenases (PNTs) [13,14]. There are two transhydrodenases in *Escherichia coli*, one soluble, the udhA which is mainly for regeneration of NADH and NADP; a membrane bound, the PNT is used for the regeneration of NAD and NADPH [15]. The PNT enzymes are composed of α and β subunits encoded by the *PntA* and *PntB* genes, respectively. Most enzymes are located in the cell membrane and they catalyze the reduction of NADP^+^ to NADPH via oxidation of NADH to NAD^+^: *NADPH* + *NAD*^+^ ⇌ *NADP*^+^ + *NADH*[16,17]. Anderlund *et al*. studied the physiological effects of interconversion between NAD(H) and NADP(H) in *S. cerevisiae* expressing membrane-bound PNTs from *E. coli* during anaerobic glucose fermentation [13]. Boonstra *et al*. successfully regenerated NAD^+^ and NADPH in a cell-free system to gain high yields of hydromorphone using the soluble PNT from *Pseudomonas fluorescens*[14].

**Figure 1:**
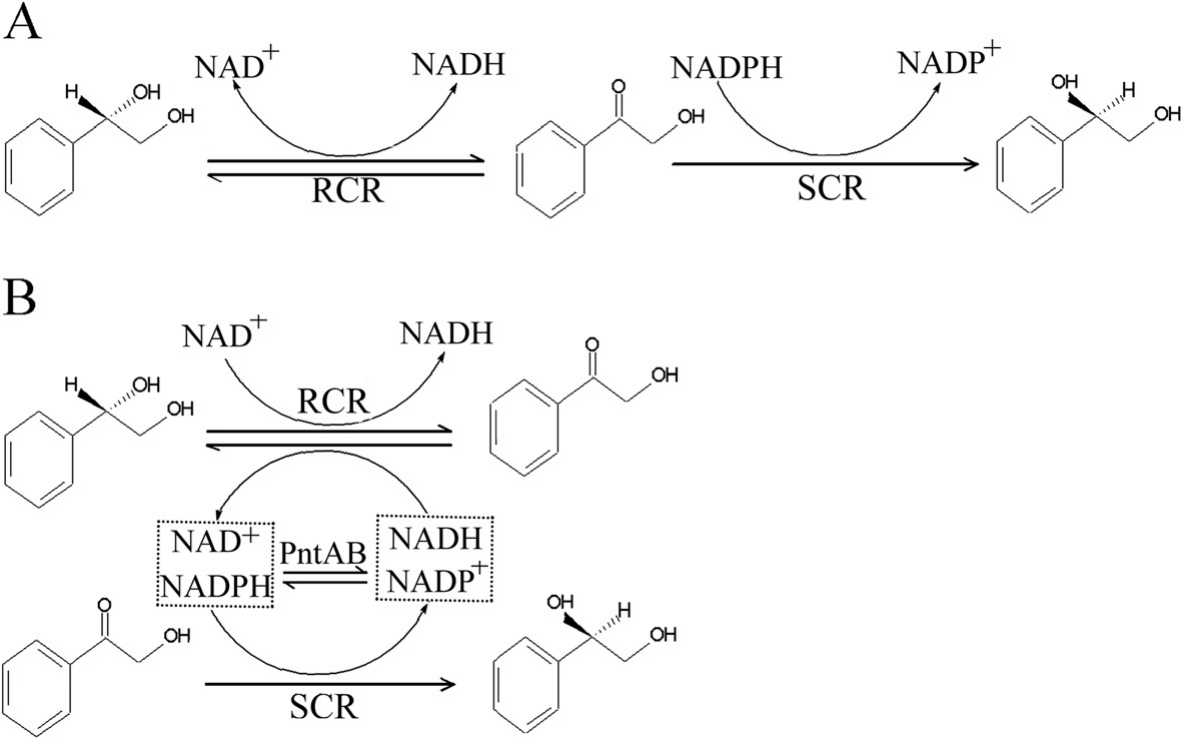
**A. Reaction catalyzed by NAD^+^-dependent RCR and NADPH-linked SCR. B. Cofactor cycling by PntA and PntB in the stereoconversion of (*S*)-PED from (*R*)-isomer.** Redox cofactors in the metabolic pathway with (*S*)-PED:(*R*)-PED are converted to the (*S*)-isomer by an NAD^+^-dependent RCR and NADPH-linked SCR from *C. parapsilosis*. The enzymes PntA and PntB from *E*. *coli* catalyze reversible interconversions between NAD(H) and NADP(H), thereby regenerating NAD^+^ and NADPH and entering the pathway with (*S*)-PED through RCR and SCR.

In our previous work, the enzymes (*R*)-carbonyl reductase (RCR) and (*S*)-carbonyl reductase (SCR) from *Candida parapsilosis* CCTCC M203011 were found to catalyze the synthesis of valuable optically active (*S*)-1-phenyl-1,2-ethanediol (PED) from the (*R*)-isomer [18-20]. The enzymatic biosynthesis included two sequential redox reactions and both cofactor couples, NAD^+^ and NADPH, are needed. First, (*R*)-PED is oxidized to the intermediate 2-hydroxyacetophenone (HAP) by NAD^+^-linked RCR, and then HAP is reduced to (*S*)-PED by NADPH-dependent SCR (Figure 1A). The RCR and SCR enzymes were expressed in *E*. *coli* at different levels, with their protein or structural characteristics becoming obvious [18-20]. The SCR enzyme had much higher catalytic efficiency than that of RCR [21]. Recently, it was found that the bioconversion of (*R*)-PED to its enantiomer by engineered *E*. *coli* co-expressing RCR and SCR is slow and gives low yields, probably as a result of insufficient cofactors or imbalanced redox cofactor ratios, resulting in unbalanced enzyme functions in microorganisms [21]. In this work, to achieve the stereoconversion of (*S*)-PED from the (*R*)-configuration efficiently, in one-step, using recombinant *E*. *coli* and simultaneous NAD^+^–NADPH regeneration *in situ*, we introduced PNT enzymes from *E*. *coli* into the metabolic pathways of RCR and SCR (Figure 1B). The effects of PNT introduction on chiral alcohol metabolism were investigated, including cell growth properties and cofactor(s) metabolic flux, and the process was optimized. This work provides a new strategy for improving the metabolic flux of chiral alcohols by the distribution of NAD(H) and NADP(H) through the introduction of heterogeneous PNTs in *E*. *coli*.

## Results and discussion

### Construction of recombinant *E. coli* harboring RCR, SCR, PntA, and PntB

In any system based on carbonyl reductase catalysis of the bioconversion of a chiral alcohol, insufficient cofactors or their unbalanced ratios results in low biotransfomation efficiency [5,7,11]. Heterogeneous expressions of PNTs (PntA and PntB) were expected to increase the overall intracellular NAD^+^ and NADPH pool, or to rebalance them, thus improving the flux of NAD^+^- and/or NADPH-dependent pathways [15].

It has been reported that the NAD^+^-linked RCR from *C. parapsilosis* reduces (*R*)-PED to HAP, and the NADPH-dependent SCR catalyzes HAP to (*S*)-PED. To prepare (*S*)-PED from the (*R*)-enantiomer efficiently in one-step, PNTs from *E. coli* were introduced into the pathway catalyzed by RCR and SCR through a “multi-enzyme approach”. Two compatible plasmids pETDuet™-1 and pACYCDuet™-1 (Novagen, Darmstadt, Germany) were used, each of which contained two multiple cloning sites. The expression plasmids pET-RS and pACYC-AB were constructed and then transformed in competent cells of *E. coli* using standard techniques, as shown in Additional file 1: Figure S1 in the supplemental materials. The recombinant RS, AB, and RSAB strains were shaped after being verified by DNA sequencing.

### Co-expression of recombinant enzymes

The recombinant strains were induced with 0.1 mM isopropyl-β-thiogalactopyranoside (IPTG) at 37°C. SDS–PAGE analysis (Additional file 1: Figure S2 in the supplemental materials) showed that two obvious bands (about 37 kDa and 31 kDa) corresponding to the sizes of RCR and SCR [18] were observed in cell-free extracts of RS and RSAB. The other two bands (about 50 kDa and 47 kDa), corresponding to the sizes of PntA and PntB, were apparent in cell extracts (without centrifugation) of AB and RSAB [6,7,13,14]. So, in the cells of RSAB, four proteins were all expressed.

The specific enzyme activities were determined in cell extracts of the recombinant strains at the exponential growth phase. The results are summarized in Table 1. The activities of RCR and SCR were 0.383 U/mg and 1.871 U/mg in the cell-free extracts of the RS strain. In the cell-free extracts of the RSAB strain, they were 0.349 U/mg and 1.758 U/mg, respectively; slightly lower than those in RS. The results suggested that the introduction of PNTs had almost no effect on the activities of the target enzymes, i.e., RCR and SCR, involved in the (*S*)-PED pathway. Most of the transhydrogenase activity was found in the cell membranes of the AB and RSAB strains. The specific activities of PntA and PntB were about 0.01 U/mg and 0.10 U/mg, respectively, in the cell-free extracts and cell membranes of the AB and RSAB strains. Sauer *et al*. also found that the membrane-bound transhydrogenase was the major one, and the low transhydrogenase activity in cell-free extracts may be caused by the isoenzymes of PNTs [15]. These results show that the four enzymes, RCR, SCR, PntA, and PntB, were all functionally expressed in the different fractions. For the whole-cell bioconversion, the location of PNTs in the cell membrane had no effect on the stereoconversion efficiency of (*S*)-PED from the (*R*)-enantiomer by engineered *E*. *coli*. The PNTs-mediated NAD^+^–NADPH regeneration system was therefore successfully introduced into the metabolic pathway of (*S*)-PED with RCR and SCR in *E*. *coli*.

**Table 1:** **Enzyme activities and stereoconversions of (*R*)-PED^a^ to (*S*)-enantiomer by recombinant *E. coli *strains**

**Strains/ plasmids**	**Specific activities (U/mg)**				**Biotransformation**	
	^**b**^**RCR**	^**c**^**SCR**	^**d**^**PNT**		**Optical purity (%e.e.)**	**Yield (%)**
			**Cell-free extracts**	**Cell membranes**		
CK	NT	NT	0.010±0.001	0.021±0.001	5.4±0.05	4.8±0.05
RS	0.383±0.017^e^	1.871±0.043	0.013±0.002	0.021±0.005	64.3±0.07	52.7±0.04
AB	NT	NT	0.014±0.004	0.112±0.002	20.7±0.13	11.5±0.09
RSAB	0.349±0.010	1.758±0.027	0.013±0.001	0.096±0.001	93.5±0.12	87.4±0.09

The stereoconversion of (*R*)-PED to (*S*)-PED was performed with the first addition of 1 mM NAD^+^ and 1 mM NADPH, at pH 6.5 and 35°C.

Notes: ^a^ PED, 1-phenyl-1,2-ethanediol;

^b^ RCR, (*R*)-carbonyl reductase, the enzyme activity was measured using (*R*)-PED as substrate;

^c^ SCR, (*S*)-carbonyl reductase, the enzyme activity was measured using 2-hydroxyacetophenone as substrate;

^d^ PNT, nicotinamide nucleotide transhydrogenase;

^e^ Mean ± standard deviation;

NT, no detectable enzyme activity.

### Rebalancing of intracellular nucleotides by introduction of PNTs

Once the PNTs were successfully introduced into the recombinant *E. coli*, we measured the intracellular concentrations of NAD^+^, NADH, NADP^+^, and NADPH, and their ratios, in the recombinant cells in the exponential growth phase. The data obtained are summarized in Table 2. Although no significant differences in total nucleotides concentrations were observed among the four strains, CK, RS, AB, and RSAB, or their early and late exponential growth phases (data not shown), the four nucleotides (NADH, NAD^+^, NADPH, and NADP^+^) had different concentrations in the different *E. coli* strains. The presence of PNTs (in the AB and RSAB strains) increased the NADH and NAD^+^ concentrations and decreased the NADPH pool, but kept NADP^+^ at a stable level, compared with the strains without PNTs (RS and CK). This resulted in an approximately two-fold decrease in the NADPH/NADP^+^ ratio and the NADH/NAD^+^ level remained constant. The most striking change was that the value of [NADPH/NADP^+^/[NADH/NAD^+^ decreased more than two-fold (from 36 to 17) in the presence of PNTs (Table 2). These experiments show that the introduction of PNTs did not change the total concentration of the four nucleotides, but their ratios were redistributed in the engineered *E. coli*. NADP^+^ and NADH are efficiently converted to NADPH and NAD^+^ by the PNTs. It was reported previously that the SCR enzyme had a much higher expression and stronger catalytic function than RCR in *E*. *coli*[21]. The increased NAD^+^ concentration and decreased NADPH pool would improve the flux of NAD^+^-mediated RCR and weaken the branch of NADPH-dependent SCR in the RSAB strain. The cofactor rebalancing was expected to redistribute the metabolic flux, contributing to the biosynthesis of the final product, (*S*)-PED.

**Table 2:** Intracellular concentrations of NAD^+^, NADH, NADP^+^, and NADPH in recombinant cells during exponential growth

**Strains**	**Intracellular concentrations (μmol/g [dry wt] of biomass) of:**					**NADH/NAD**^**+**^**ratio**	**NADPH/NADP**^**+**^**ratio**
	**NAD**^**+**^	**NADP**^**+**^	**NADH**	**NADPH**	**Total**		
CK	1.78±0.05	0.13±0.02	0.28±0.01	0.74±0.04	2.93±0.05	0.16±0.02	5.69±0.02
RS	1.71±0.08	0.11±0.01	0.31±0.01	0.68±0.07	2.81±0.08	0.18±0.02	6.18±0.02
AB	2.14±0.04	0.17±0.01	0.34±0.02	0.39±0.06	3.04±0.04	0.16±0.02	2.29±0.02
RSAB	2.19±0.02	0.15±0.01	0.37±0.02	0.42±0.02	3.13±0.12	0.17±0.01	2.80±0.02

### Distribution of intracellular nucleotides had little effect on cell growth

**Figure 2:**
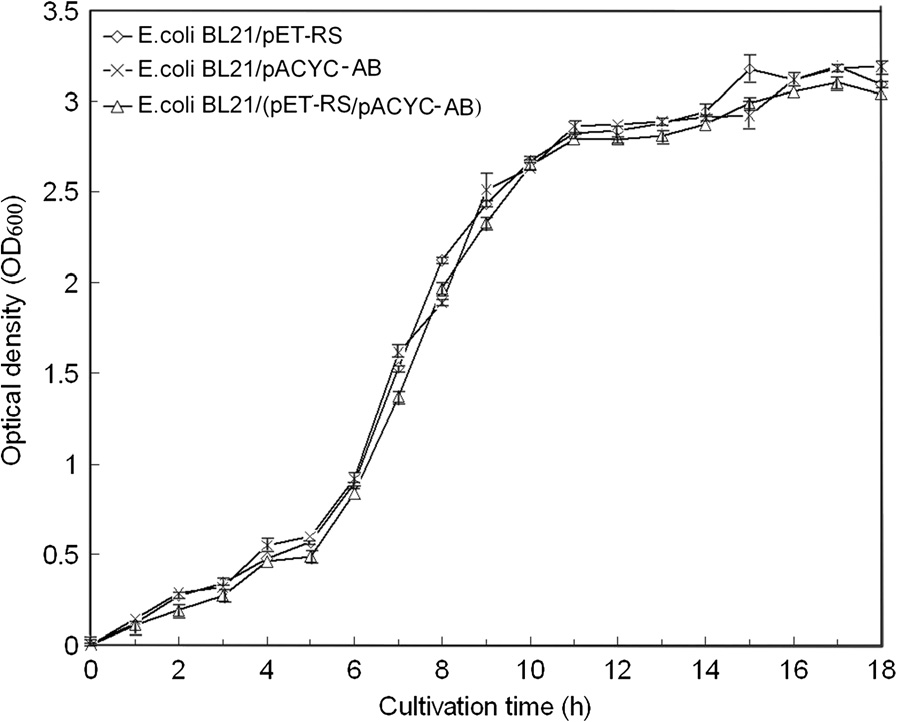
**Growth curves of RS, AB, and RSAB.** The engineered strains were cultivated at 37°C in 2.5-L flask bottles, with an initial working volume of 1.0 L, and then 1.0 mM IPTG was added at 5 h to induce protein expression. Error bars represent standard deviations (*n* = 3).

The two systems NAD^+^/NADH and NADP^+^/NADPH have separate and distinct metabolic roles [22], so the distribution of intracellular nucleotides resulting from the introduction of PNTs may affect *E. coli* cell growth. The fermentation characteristics of the recombinant *E*. *coli* were compared with data from flask experiments. Cell growth was determined by measuring the turbidity of the culture at *OD*_600_ using a UV–visible spectroscopy system (Agilent 8453, Germany). Based on the three-stage division of cell growth curves (Figure 2), the engineered RSAB strain expressing four enzymes (RCR, SCR, PntA, and PntB) grew at a similar rate compared with RS and AB. The results suggested that the introduction of PNTs into *E. coli* had almost no effect on cell growth properties, and the distribution of nucleotide concentrations in *E. coli* did not inhibit cell growth. As Zhang *et al*. reported, a constant level of total nucleotides was the key factor for cell growth properties [23]. A multi-enzyme system based on RCR, SCR, PntA, and PntB catalyzing the desired bioconversion of (*S*)-PED from the (*R*)-isomer, plus cofactor regenerations, would be preferable for further studies of enzyme-catalyzed reactions.

### One-step stereoconversion of (*R*)-PED to (*S*)-enantiomer plus NAD^+^–NADPH *in*-*situ* regeneration using the RSAB strain

Using the recombinant *E. coli* cells as the catalyst, the effects of the rebalancing of cofactors by the introduction of PNTs on the stereoconversion of (*R*)-PED to the (*S*)-isomer were investigated at pH 6.5, as a compromise among the pH optima of the four enzymes (pH 6.0-7.0 for PntA and PntB [13,24], pH 6.0 for RCR [25], and pH 5.0 for SCR [18]), with an initial addition of 1 mM NAD^+^ and 1 mM NADPH. To facilitate transport of the cofactors, 1% toluene was used to increase cell permeability, so that the reaction compounds as well as the coenzymes can pass through the membranes more easily [26]. Samples were taken and analyzed with respect to formation of (*S*)-PED after specific times. The results (Table 3) showed that all the strains produced (*S*)-PED with different transformation efficiencies: the RS strain produced (*S*)-PED with a low optical purity (64.3%) and yield (52.7%), whereas the RSAB strain produced (*S*)-PED with a high optical purity of 93.5% and a high yield of 87.4%. Although the strains AB and CK had no target enzymes (RCR and SCR) catalyzing (*S*)-PED production, they also exhibited weak bioconversion, and required the same time (48 h) to achieve the highest efficiency with *E*. *coli*–SCR (Table 3) [18]. Importantly, the asymmetric reaction using RSAB proceeded most quickly among the four strains and reached the highest point at 6 h, suggesting that the cofactor redistribution by the introduction of PNTs accelerated the transformation of (*S*)-PED from the (*R*)-isomer. Efficient one-step stereoconversion of (*R*)-PED to the (*S*)-enantiomer plus NAD^+^–NADPH *insitu* regeneration was therefore achieved using the RSAB strain.

**Table 3:** **Bioconversions of (*R*)-PED to (*S*)-isomer with RSAB cells for different NAD^+^/NADPH ratios**

	**No cofactors**	**Only NAD**^**+**^	**Only NADPH**	**NAD**^**+**^**: NADPH**						
				**1 : 1**	**1 : 2**	**1 : 3**	**1 : 4**	**2 : 1**	**3 : 1**	**4 : 1**
Optical purity (% e.e.)	15.7±0.03	30.2±0.02	24.8±0.04	93.5±0.12	81.2±0.08	76.3±0.10	71.9±0.04	95.4±0.06	97.4±0.11	97.3±0.07
Yield (%)	8.9±0.05	21.8±0.03	16.1±0.02	87.4±0.09	70.3±0.02	67.5±0.02	62.0±0.03	90.1±0.02	95.2±0.02	95.3±0.04

The stereoconversion of (*R*)-PED to (*S*)-isomer was performed in the presence of various ratios between NAD^+^ (1.0 – 2.0 mM) and NADPH (1.0 – 2.0 mM), with both NAD^+^ and NADPH or with neither, at pH 6.5 and 35°C.

### Improving stereoconversion efficiency by optimizing ratio of cofactors

To improve the stereoconversion efficiency, the whole-cell biotransformation was investigated in the presence of various ratios of NAD^+^ (1.0–2.0 mM) and NADPH (1.0–2.0 mM), with both NAD^+^ and NADPH, and with neither. The optical purity and yield of (*S*)-PED was measured when the substrate (*R*)-PED concentration was 100 mM and the incubation of the reaction mixture lasted for 6 hours. As shown in Table 3, even if the initial addition of NAD^+^ and/or NADPH was omitted, the reaction proceeded as a result of the natural existence of cofactors in *E. coli* cells. However, the biotransformation efficiency was very low. If neither of the cofactors was added, the optical purity and yield of (*S*)-PED were at least three times lower than when both cofactors were present. In the case of NADPH, the biotransformation was less than that with NAD^+^, which suggested that improving the metabolic flux of NAD^+^-mediated RCR would be more beneficial for the biotransformation efficiency than strengthening the branch of NADPH-dependent SCR. (*S*)-PED was efficiently produced if both NAD^+^ and NADPH were present in the reaction mixture, but different NADH/NADP^+^ ratios resulted in different biotransformation efficiencies. When the NAD^+^/NADPH ratio was lower than 0.5, the optical purities and yields were not satisfactory. When the ratio was 3.0 or higher, the optical purities and yields of (*S*)-PED reached the highest levels, i.e., about 97% and 95%, respectively. Compared with the RS strain, RSAB expressing PNTs stimulated an increase of 51.5% and 80.6% in the optical purity and yield of (*S*)-PED, while simultaneously reducing the substrate-use time seven-fold.

Since the steps in the reactions catalyzed by RCR or SCR occurred in a specific order (i.e., the free enzyme was first combined with the coenzyme and then with the substrate) [27], the addition of cofactors could seriously affect the enzymatic efficiency. Furthermore, the SCR enzyme had a much higher expression level and a much stronger enzymatic function than those of RCR in *E. coli*[21]. The moderate increase in NAD(H) could strengthen the RCR function, and improve the balance between NAD^+^-dependent RCR and NADP(H)-linked SCR in the enzyme-coupled system. These results suggested that the NAD(H)-mediated or NADP(H)-dependent fluxes are partly determined by their cofactor availability, and can be improved by rebalancing the metabolic branches [28], i.e., strengthening or weakening their cofactor regeneration systems [29,30].

## Conclusions

Using a multi-coenzyme approach, PNT enzymes, involved in cofactor regeneration, were introduced into the metabolic pathway, driving it in the direction of (*S*)-PED production, in the RSAB strain. The introduction of PNT enzymes resulted in slightly higher intracellular NAD(H) concentrations and a much lower NADPH pool, with cell growth and total nucleotides unaffected. The cofactor balancing of (*S*)-PED pathways caused a sharply reduced NADPH/NADP^+^ ratio but maintained the NADH/NAD^+^ ratio at a constant level during the exponential growth phase. The efficient one-step production of (*S*)-PED from the (*R*)-enantiomer, plus NAD^+^–NADPH *insitu* regeneration, was achieved by the introduction of PNTs. When the ratio of NAD^+^/NADPH was three or higher, PNT introduction resulted in significant increases in the optical purity and yield of (*S*)-PED. The results demonstrate that in certain microbial systems the cofactor-dependent (*S*)-PED pathway is not only controlled by the availability of target enzymes (RCR and SCR), but is also determined by the amount of cofactor and the ratio of reduced forms to oxidized forms [12,29]. This work provides a new strategy for preparing (*S*)-PED efficiently, using cofactor rebalancing to engineer chiral alcohol pathways by the introduction of PNT enzymes.

## Methods

### Microorganisms and chemicals

*C. parapsilosis* CCTCC M203011, obtained from the American Type Culture Collection (ATCC, USA), was used as the DNA donor of the SCR gene (*scr*). The organisms were cultivated as described previously [18,19]. *E. coli* K12 was used as the DNA donor of pyridine nucleotide transhydrogenase A and B genes (*PntA* and *PntB*). *E. coli* BL21 (DE3) and JM109 were used as host cells for gene cloning and expression experiments. *E. coli* cells were cultured at 37°C in Luria–Bertani (LB) medium, supplemented with ampicillin (100 μg/mL) and/or chloramphenicol (34 μg/mL) when necessary. After starting the cultivation, IPTG (0.1 mM) was added to the medium at 5 h. The cultures were shaken for 8 h at 37°C and harvested by centrifugation after addition of IPTG.

The enzymes and cofactors were purchased from the Sigma-Aldrich Chemical Co., Inc. The restricted enzymes, vectors, and marker DNA used for cloning and the expression experiments were purchased from Qiagen (Germany), Takara-Bio (Kyoto, Japan), Novagen (Germany), and New England Biolabs (USA). All other chemicals were of the highest grade that could be obtained commercially.

### Clonings

Genomic DNA was isolated using a Biospin Cell Genomic DNA Extraction Kit (Bioer Technology Co.). The oligonucleotide primers were designed based on the gene sequences in Table 4. The *scr* genes (GenBank ID: DQ295067) were amplified from the *C. parapsilosis* genome. The RCR gene (*rcr*) (DQ675534) was cloned using pQE-mRCR as the DNA template [18]. The *PntA* and *PntB* (NG1470 and NG1472) genes were generated from *E. coli* K12 chromosomal DNA. The PCR-amplified products were ligated to pMD18-T (Takara-Bio, Kyoto, Japan) to obtain pMD-RCR, pMD-SCR, pMD-PntA, and pMD-PntB plasmids, which were then transformed in *E. coli* JM109 cells and verified by DNA sequencing*.*

**Table 4:** Plasmids, strains and primers used

**Plasmids, strains, primers**	**Description**	**Sources**
**Plasmids**		
pMD18-T	2.7 kb, Amp^r^	Takara
pMD18-RCR	3.7 kb, pMD18-T containing *rcr*, Amp^r^	Novagen
pMD18-SCR	3.5 kb, pMD18-T containing *scr*, Amp^r^	This work
pMD-PntA	4.2 kb, pMD18-T containing *PntA*, Amp^r^	This work
pMD-PntB	4.1 kb, pMD18-T containing *PntB*, Amp^r^	This work
pETDuet™-1	5.4 kb, contains two multiple cloning sites, Amp^r^	Novagen
pACYCDuet™-1	4.0 kb, contains two multiple cloning sites, Cm ^r^	Novagen
pET-RS	7.4 kb, pETDuet™-1 containing *rcr* and *scr*, Amp^r^	This work
pACYC-AB	6.8 kb, pACYCDuet™-1 containing *PntA* and *PntB*, Cm ^r^	This work
**Strains**		
*C. parapsilosis* CCTCC M203011	DNA donors of *rcr* and *scr* genes	This laboratory
*E. coli* K12	DNA donors of *PntA* and *PntB* genes	This laboratory
*E. coli* JM109	*recA*1 *supE*44 *endA*1 *hsdR*17 *gyrA*96 *relA*1 *thi*△(*Lac*-*proAB*)F’	This laboratory
*E. coli* BL21(DE3)	F^-^*ompT hsdS*_*B*_(*r*_*B*_^-^*m*_*B*_^-^) *gal dcm* (DE3)	Novagen
CK	*E*. *coli* BL21 bearing pETDuet™-1 and pACYCDuet™-1	
RS	*E*. *coli* BL21 bearing pET-RS	This work
AB	*E*. *coli* BL21 bearing pACYC-AB	This work
RSAB	*E*. *coli* BL21 bearing pET-RS and pACYC-AB	This work
**Primers**	**5′ → 3′**	
RCR_F	ATCGATCGCATATGTCAATTCCATCAAGCCAGTACGG(*NdeI*)	This work
RCR_R	TGACTCTCGAGCTATGGATTAAAAACAACACGACC(*Xho*I)	This work
SCR_F	ATCGAATTCGATGGGCGAAATCGAATCTTATTG(*Eco*RI)	This work
SCR_R	TGACTGCGGCCGCCTATGGACACGTGTATCCACCGTC(*Not*I)	This work
PntA_F	CGCGGATCCATGCGAATTGGCATACCAAG (*Bam*HI)	This work
PntA_R	CCCAAGCTTTTAATTTTTGCGGAACATTTTC (*Hin*dIII)	This work
PntB_F	CGCGATATCATGTCTGGAGGATTAGTTAC (*Eco*RV)	This work
PntB_R	CCCCTCGAGTTACAGAGCTTTCAGGATTG (*Xho*I)	This work

### Construction of co-expression systems

To co-express *rcr*, *scr*, *PntA*, and *PntB* in *E. coli* BL21 (DE3), two compatible plasmids, pETDuet™-1 and pACYCDuet™-1 (Novagen), were used. The plasmids pMD-RCR and pMD-SCR were digested with *Nde* I/*Xho* I and *Eco*R I/*Not* I, respectively, and then ligated into pETDuet™-1 in sequence to construct pET-RS. The fragments of *PntA* (*Bam*H I/*Hin*d III) and *PntB* (*Eco*R V/*Xho* I) were successively inserted into the corresponding sites of pACYCDuet™-1 to obtain pACYC-AB. The construction of pET-RS and pACYC-AB is shown in Additional file 1: Figure S1 in the supplemental materials. They were then introduced into *E. coli* BL21 cells individually or simultaneously. The positive strains RS, AB, and RSAB were obtained. The plasmids and strains containing different antibiotic resistances and the primers used in this study are listed in Table 4.

### Preparation of cell-free extracts

The cultured *E. coli* BL21 cells were harvested by centrifugation, suspended in 20 mM Tris–HCl (pH 8.0) and 150 mM NaCl, and then disrupted with an ultrasonic oscillator (Insonater 201 M; Kubota, Japan). After centrifugation (16 000 rpm × 40 min) at 4°C, the cell-free extracts were used for the enzyme assays. Preparation of the cell membranes of *E*. *coli* BL21 was performed as described by Clarke *et al*. [16].

### Enzyme assay

The enzymatic activities of RCR for oxidation of (*R*)-PED were measured at 35°C and pH 9.0 by spectrophotometrically recording the rate of change of NADH absorbance at 340 nm. The SCR activities for reduction of HAP were assayed at 340 nm by monitoring the change in NADPH. One unit of enzyme activity is defined as the amount of enzyme catalyzing the reduction/oxidation of 1 μmol of NAD(P)H per minute under the measurement conditions. The specific activity is the number of enzyme units per milligram. The standard assays were performed as described by Nie *et al*. [18].

Because one isoform is a membrane-bound protein, the PNT activities were determined in cell extracts without centrifugation [31]. Briefly, the enzyme activity was measured spectrophotometrically for 1 min at 30°C at 375 nm, using 3-acetylpyridine adenine nucleotides, and 10–100 μL crude cell extracts, as described by Rydström *et al*. [23]. Protein concentrations were measured using the method described by Bradford [32].

### Biotransformations and analytical methods

Using washed cells of RS, AB, and RSAB as the catalysts, the reaction was carried out as described previously [33]. When necessary, 1% toluene was added to the reaction mixture [18]. The (*S*)-PED product was extracted with ethyl acetate, and the organic layer was used for analysis. The optical purities and yields of (*S*)-PED were determined using high-performance liquid chromatography on a Chiralcel OB-H column (Daicel Chemical Ind. Ltd., Japan). All plots were shown as the means of three independent experiments.

### Determination of intracellular nucleotide concentrations

The extraction of intracellular nucleotides was carried out as previously described by Nissen *et al*. [28], with minor modifications. The collected 5.0 mL of recombinant *E*. *coli* culture was mixed with 20 mL of 60% methanol (−40°C) within 1 s. A 50 mM KPO_4_ buffer (pH 5.0) and 50 mM Tris–HCl (pH 9.0) were used for extraction of NAD^+^/NADP^+^ and NADH/NADPH, respectively. The nucleotide concentrations were measured immediately after reducing the sample volumes by evaporation under vacuum (30 min, 5°C). The contents of NAD^+^, NADH, NADP^+^, and NADPH in the samples, obtained by cold methanol extraction, were determined as described by Hu [34], using standard curves for each compound. Assays were performed in triplicate.

## Abbreviations

HAP: 2-hydroxyacetophenone; PntA: Pyridine nucleotide transhydrogenase subunit α; *PntA*: Pyridine nucleotide transhydrogenase A gene; PntB: Pyridine nucleotide transhydrogenase subunit β; *PntB*: Pyridine nucleotide transhydrogenase B gene; PED: 1-phenyl-1,2-ethanediol; RCR: (*R*)-carbonyl reductase; *rcr*: (*R*)-carbonyl reductase gene; SCR: (*S*)-carbonyl reductase; *scr*: (*S*)-carbonyl reductase gene.

## Competing interests

The authors declare that they have no competing interests.

## Authors' contributions

RZ performed all experiments except of the bioreactor cultivations, and drafted the manuscript. YX conceived and managed the projects. RX helped with the experimental implementations and supplied good suggestions on revising the manuscript. BZ and LW helped with the microbial cultivations and biotransformation. All authors read and approved the final manuscript.

## Supplementary Material

Additional file 1 **Figure S1.**Strategy of co-expression plasmid construction. **Figure S2**. SDS-PAGE analysis cell extracts of *E*. *coli* transformants with or without centrifugation. Lanes 1, RS; Lanes 2, AB; Lanes 3, CK; 4, RSAB; Lane M, molecular mass markers. The gel was stained for protein with Coomassie Brilliant Blue R-250. Click here for file
