# Supplementary material for: Efficient one-step production of (S)-1-phenyl-1,2-ethanediol from (R)-enantiomer plus NAD+–NADPH in-situ regeneration using engineered Escherichia coli
Source: Microb Cell Fact. 2012 Dec 29;11:167. doi: 10.1186/1475-2859-11-167 (PMC3551732; doi:10.1186/1475-2859-11-167)

**Efficient one-step production of (*S*)-1-phenyl-1,2-ethanediol from (*R*)-enantiomer plus NAD^+^-NADPH *in*-*situ* regeneration using engineered *Escherichia coli***

Rongzhen Zhang^1,2^, Yan Xu^1,2^*, Rong Xiao^3^, Botao Zhang^1,2^, Lei Wang^1,2^

Rongzhen Zhang (rzzhang666@yahoo.com.cn)

Yan Xu (biosean@yahoo.com.cn)

Rong Xiao (r_xiao@yahoo.com)

Botao Zhang ([liulangdeyeshui@163.com](mailto:liulangdeyeshui@163.com))

Lei wang (173890119@qq.com)

^1^ Key Laboratory of Industrial Biotechnology of Ministry of Education & School of Biotechnology, Jiangnan University, Wuxi 214122, P. R. China

^2^ National Key Laboratory for Food Science, Jiangnan University, Wuxi 214122, P. R. China

^3^ Center for Advanced Biotechnology and Medicine, Rutgers University, Piscataway, NJ 08854, USA

***Corresponding author: Yan Xu**

**Tel: +86-510-85918201; Fax: +86-510-85864112**

***Email address*:** [**biosean@yahoo.com.cn**](../biosean@yahoo.com.cn)

**Present address: School of Biotechnology, Jiangnan University, 1800 Lihu Avenue, Wuxi, 214122, P. R. China**

**Supplemental materials**

**Figure S1.** [Strategy of co-expression plasmid construction](http://www.springerimages.com/Images/Chemistry/1-10.1007_s00253-008-1428-z-0)


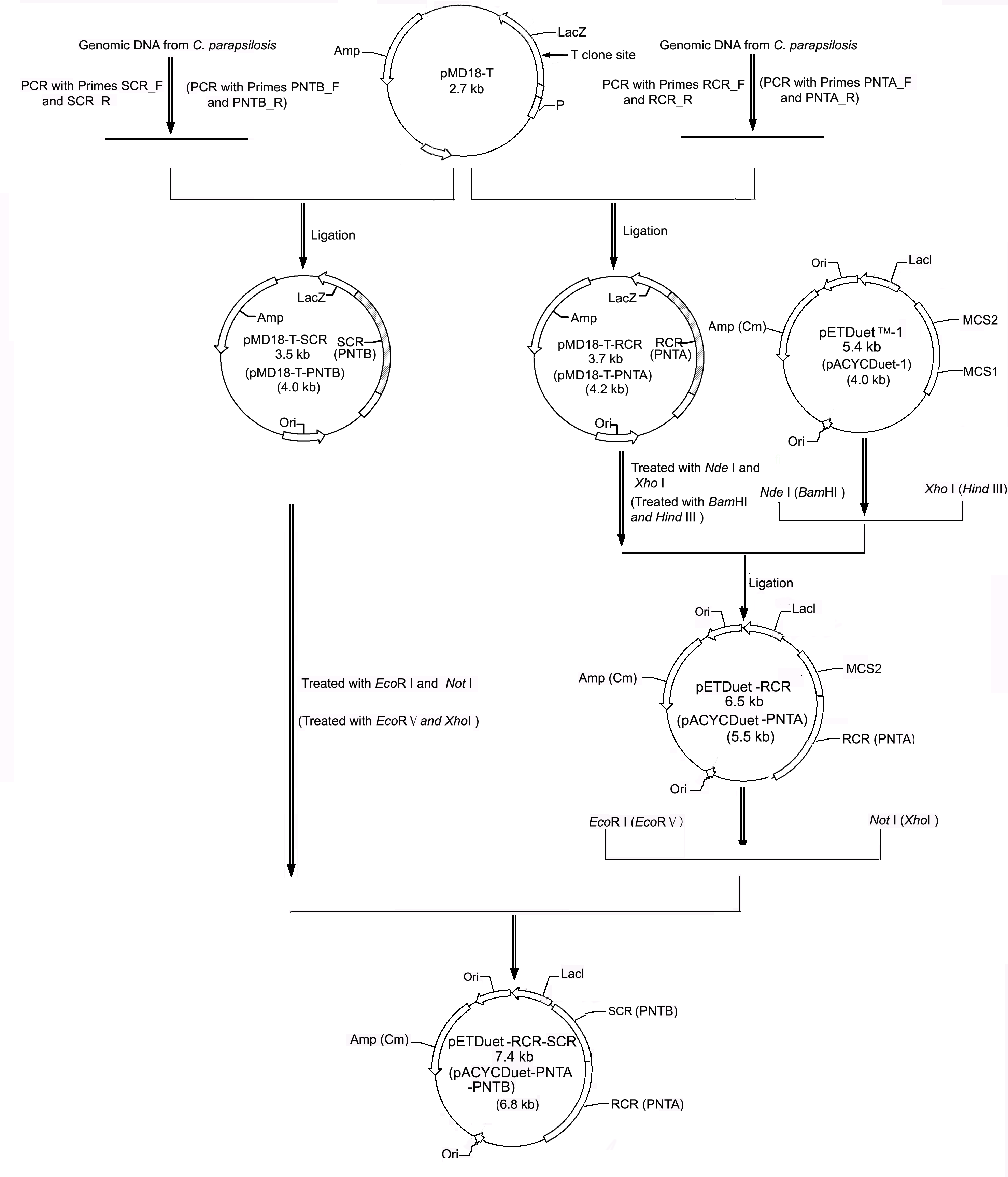


**Figure S2.** SDS-PAGE analysis cell extracts of *E*. *coli* transformants with or without centrifugation. Lanes 1, RS; Lanes 2, AB; Lanes 3, CK; 4, RSAB; Lane M, molecular mass markers. The gel was stained for protein with Coomassie Brilliant Blue R-250．


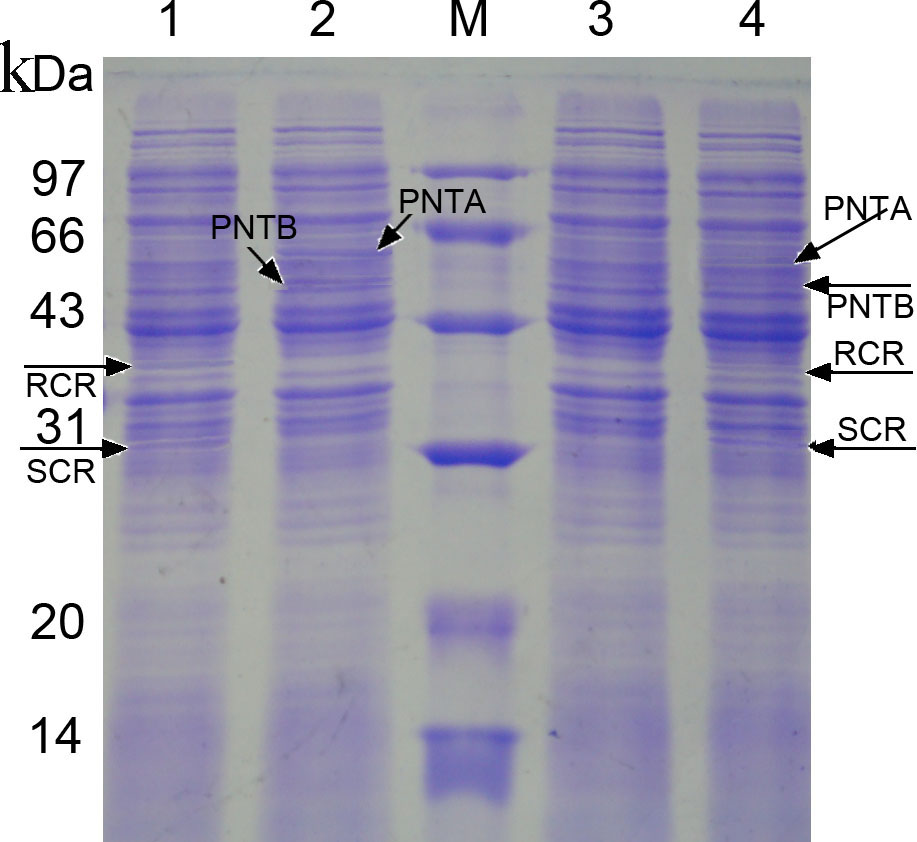

Supplement: Additional file 1 — Figure S1.Strategy of co-expression plasmid construction. Figure S2. SDS-PAGE analysis cell extracts of E. coli transformants with or without centrifugation. Lanes 1, RS; Lanes 2, AB; Lanes 3, CK; 4, RSAB; Lane M, molecular mass markers. The gel was stained for protein with Coomassie Brilliant Blue R-250. [file 1475-2859-11-167-S1.doc]
